# Thermal expansion coefficient of few-layer MoS_2_ studied by temperature-dependent Raman spectroscopy

**DOI:** 10.1038/s41598-021-86479-6

**Published:** 2021-03-29

**Authors:** Zhongtao Lin, Wuguo Liu, Shibing Tian, Ke Zhu, Yuan Huang, Yang Yang

**Affiliations:** 1 Beijing National Laboratory for Condensed Matter Physics, Institute of Physics, Chinese Academy of Sciences, P O Box 603, Beijing, 100190 People’s Republic of China; 2 Faculty of Materials and Manufacturing, Beijing University of Technology, Beijing, 100124 People’s Republic of China; 3 Department of Materials Science and Engineering, and State Key Laboratory of Heavy Oil Processing, College of Science, China University of Petroleum Beijing, No. 18 Fuxue Rd., Beijing, 102249 People’s Republic of China

**Keywords:** Two-dimensional materials, Raman spectroscopy

## Abstract

The thermal expansion coefficient is an important thermal parameter that influences the performance of nanodevices based on two-dimensional materials. To obtain the thermal expansion coefficient of few-layer MoS_2_, suspended MoS_2_ and supported MoS_2_ were systematically investigated using Raman spectroscopy in the temperature range from 77 to 557 K. The temperature-dependent evolution of the Raman frequency shift for suspended MoS_2_ exhibited prominent differences from that for supported MoS_2_, obviously demonstrating the effect due to the thermal expansion coefficient mismatch between MoS_2_ and the substrate. The intrinsic thermal expansion coefficients of MoS_2_ with different numbers of layers were calculated. Interestingly, negative thermal expansion coefficients were obtained below 175 K, which was attributed to the bending vibrations in the MoS_2_ layer during cooling. Our results demonstrate that Raman spectroscopy is a feasible tool for investigating the thermal properties of few-layer MoS_2_ and will provide useful information for its further application in photoelectronic devices.

## Introduction

Two-dimensional (2D) transition metal dichalcogenides (TMDs), especially monolayer 2D TMDs, have attracted enormous attention in the past decade because of not only their striking physical properties^1,2^ but also their potential applications in electronic, photonic and thermoelectric devices^3–6^. However, obtaining large-area single crystalline monolayer 2D TMDs is still challenging, hindering their applications in devices. Compared with monolayer 2D TMDs, few-layer 2D TMDs are much easier to achieve by physical or chemical methods. In recent years, few-layer 2D TMDs have received increasing attention due to their interesting physical properties and applications in electronic and optoelectronic devices^7–11^. Neri and coauthors reported the strain induced semiconductor–metal transition in few-layer MoS_2_^7^. High-speed vertical photodiodes based on few-layer MoS_2_ have been fabricated using asymmetric metal contacts, exhibiting an external quantum efficiency of up to 7%^9^. A simple few-layer MoS_2_-based photodetector employing vertical Schottky junctions of Au-MoS_2_-indium tin oxide (ITO) was proposed by Gong et al.^11^. It has been demonstrated that the physical properties of MoS_2_ can be significantly affected by the interactions between MoS_2_ and the substrate, which causes strain, doping and defects^12–16^. One of the prominent substrate effects is the strain created on the MoS_2_ layer due to the difference in binding energies and the lattice mismatch between the substrate and MoS_2_. Additionally, the electronic structure of MoS_2_ can be modulated by external strain, and the PL emission of MoS_2_ will change as a result^16–18^.

The self-heating effect occurs while a device is working under a current flow or light irradiation, so the thermal properties of few-layer MoS_2_ are important criteria that affect the performance of related electronic and optical devices. For example, the thermoelectric energy conversion ability of MoS_2_ is related to the low thermal conductivity^19^, whereas high performance of electronic devices requires high thermal conductivity^20^. Alongside the thermal conductivity and thermal transport properties, the thermal expansion coefficient is another important thermal property of MoS_2_. The thermal expansion coefficient (TEC) mismatch between the substrate and MoS_2_ introduces additional internal strain to the MoS_2_ layer. Consequently, the optical and electronic performances of MoS_2_ devices supposedly change due to the thermal strain. Therefore, clear insight into the TEC of MoS_2_, especially the TEC mismatch between MoS_2_ and the substrate, is a key for studying the thermal stability and intrinsic optical properties of few-layer MoS_2_-based devices.

Raman spectroscopy has been demonstrated to be a versatile tool for investigating 2D TMDs^21–23^. In the past decade, the temperature effects on the Raman modes of 2D TMDs have been widely investigated^24–29^. However, the temperature behaviors of the Raman peaks of TMDs are still controversial. Some researchers reported that the peak positions varied linearly with increasing temperature^24–27^. Late et al*.* reported that both monolayer and few-layer MoSe_2_ and WSe_2_ exhibit a linear temperature dependence^25^. A linear temperature dependence of Raman modes was also observed in monolayer Mo_1−x_W_x_S_2_^27^. In recent years, some studies have demonstrated that the temperature dependence of the Raman peak positions for TMDs can be fitted by a nonlinear function. Su and coauthors employed temperature-dependent Raman spectroscopy to study the substrate bonding effects on MoS_2_ and WS_2_ and expressed the temperature dependence of Raman modes using a third-order polynomial function^28–30^. Although the reported temperature dependences of Raman modes for 2D TMDs differ, the TEC mismatch between TMDs and substrates is widely accepted to play an important role in the temperature evolution of Raman modes. To eliminate the substrate effects, suspended TMDs have been used to study the intrinsic properties of TMDs in recent years^31–36^. Two-ten times improvement of the mobility and on/off ratio was observed in suspended monolayer MoS_2_^31^. The elastic coefficients, including the 2D elastic modulus and Young’s modulus, were obtained for suspended multilayer WSe_2_^32^. Moreover, the intrinsic thermal conductivity has been investigated for monolayer and few-layer MoS_2_^33,34^. However, to our knowledge, the TEC mismatch effect in few-layer MoS_2_ has not been systematically studied, and the intrinsic TEC of few-layer MoS_2_ has not yet been obtained.

In this work, suspended MoS_2_ and supported few-layer (2–6 layer) MoS_2_ was comprehensively investigated using Raman spectroscopy in the temperature range from 77 to 557 K. The temperature dependence of suspended MoS_2_ exhibited different trends from that of supported few-layer MoS_2_, which could be attributed to the TEC mismatch between MoS_2_ and the substrate. Moreover, the temperature dependence of the Raman modes varied with the number of layers. Removing the substrate effect by adopting suspended MoS_2_ as a freestanding MoS_2_, the intrinsic TECs of few-layer MoS_2_ was obtained. Prominent differences between our results and previous reports were observed and discussed in detail.

## Results and discussion

**Figure 1:**
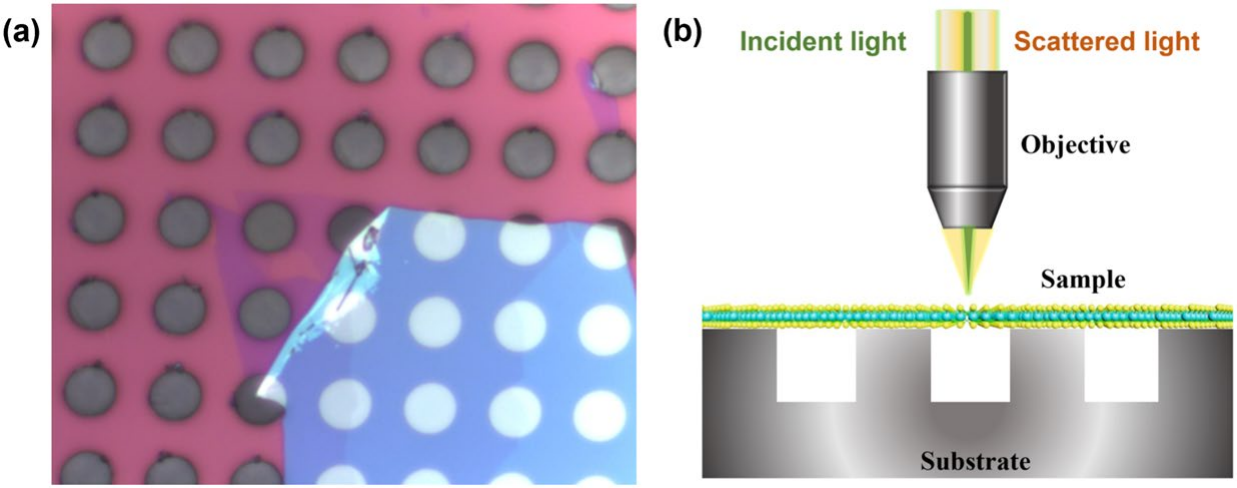
(**a**) Optical image of 2-layer MoS_2_ on a prepatterned SiO_2_/Si substrate with a 5 μm hole array. (**b**) Schematic illustration of Raman measurement for MoS_2_ suspended on microholes.

The suspended and supported samples were fabricated by transferring MoS_2_ with 2–6 layers onto microholes, which were prepared using a modified mechanical exfoliation method^37^ (see “Methods”). Figure 1a presents the optical microscopy image of suspended 2-layer MoS_2_ as an example.

**Figure 2:**
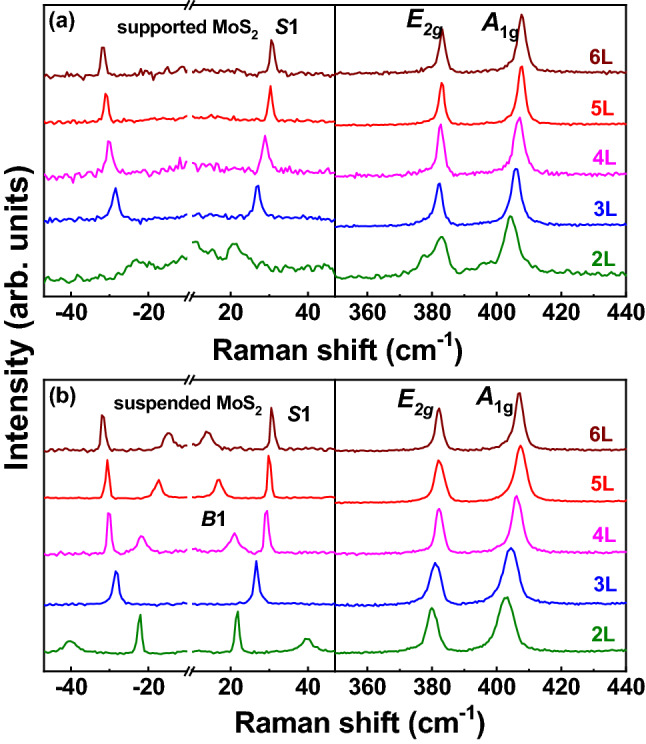
Raman spectra of (**a**) supported and (**b**) suspended MoS_2_ with different numbers of layers collected at room temperature.

Raman spectra were collected using a confocal micro-Raman spectrometer (Horiba Evolution) under backscattering geometry, as exhibited in Fig. 1b. Figure 2 presents the room-temperature Raman spectra of suspended and supported few-layer MoS_2_, which exhibit the typical spectral features of MoS_2_ previously reported^22,23^. Two high frequency peaks appear at approximately 380 cm^−1^ and 405 cm^−1^, originating from the lattice vibration of bulk MoS_2_. Due to the crystalline symmetry changes between bulk, monolayer and few-layer MoS_2_, the symmetric representations of these two Raman modes are different. For convenience, these two modes are identified as the E_2g_ and A_1g_ modes, respectively, following the assignments for bulk MoS_2_^23^. The bulk-vibrational Raman modes shift to higher positions with increasing number of layers. In recent years, ultralow frequency (ULF) Raman spectroscopy has attracted the interest of more researchers because it has been demonstrated to be a feasible tool for studying the interlayer vibrational modes of TMDs^38–40^. The ULF Raman peaks originate from the in-plane (shear) and out-of-plane (breathing) vibrations of MoS_2_, which have been used to identify the number of MoS_2_ layers^39,40^. The sharp peak is denoted as a shear mode (S1), while the broad peak is assigned as a breathing mode (B1), as shown in Fig. 2. Notably, the signal-to-noise ratio (SNR) of the Raman peaks, especially the ULF Raman peaks, of suspended MoS_2_ is much better than that of supported MoS_2_, and more detailed spectral information can be clearly seen. Both the S1 and B1 modes are clearly observed on suspended MoS_2_, whereas only the S1 mode is detected on supported MoS_2_. The MoS_2_ layer is pinned on the substrate through van der Waals forces. The dielectric environment created from by the substrate may have effect on the local electromagnetic field due to the multiple reflection inside the monolayer^16^. The enhanced Raman signal of the suspended MoS_2_ can be attributed to the isolation from the substrate effect^35^. Moreover, the E_2g_ mode for the supported 2L MoS_2_ is asymmetric, as shown in Fig. 2b. As Mignuzzi et al. reported, defects could induce not only an asymmetric line shape but also Raman peaks arising from zone-edge phonon modes^41^. In our work, no additional peak was observed in the spectrum for supported 2L MoS_2_, suggesting that the strain is the dominant effect rather than defects. The E_2g_ mode of monolayer MoS_2_ has been demonstrated to split into two singlets as the external strain is increased^42,43^. As presented in Fig. S1b, the E_2g_ mode can be well fitted using two peaks, which can be attributed to the strain introduced by the substrate-MoS_2_ interaction. We assume that the substrate-induced strain is the same for all the supported MoS_2_ flakes. Therefore, the strain effect on the supported 2L MoS_2_ is the most obvious because 2L MoS_2_ is thinner than the other samples.

**Figure 3:**
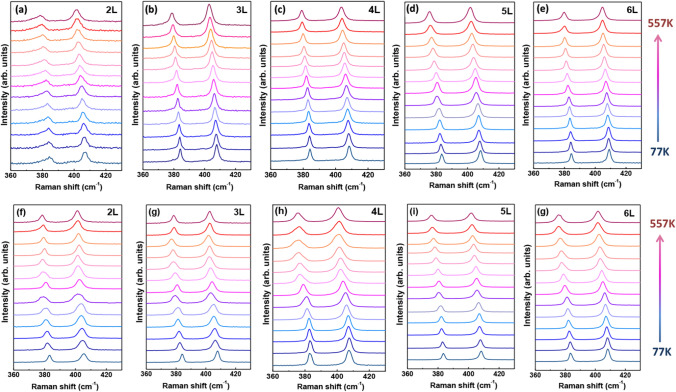
Raman spectra of (**a**–**e**) supported and (**f**–**g**) suspended few-layer MoS_2_ for different temperatures.

To deeply investigate the substrate effect, supported MoS_2_ and suspended few-layer MoS_2_ were studied in the temperature range of 77 K–557 K, and the results are displayed in Fig. 3. Prominent redshift and broadening of Raman peaks are noted for both suspended and supported MoS_2_ with increasing temperature, as exhibited in Fig. 3. These phenomena can be attributed to the thermal expansion of the crystal lattice of MoS_2_^26,27^.

**Figure 4:**
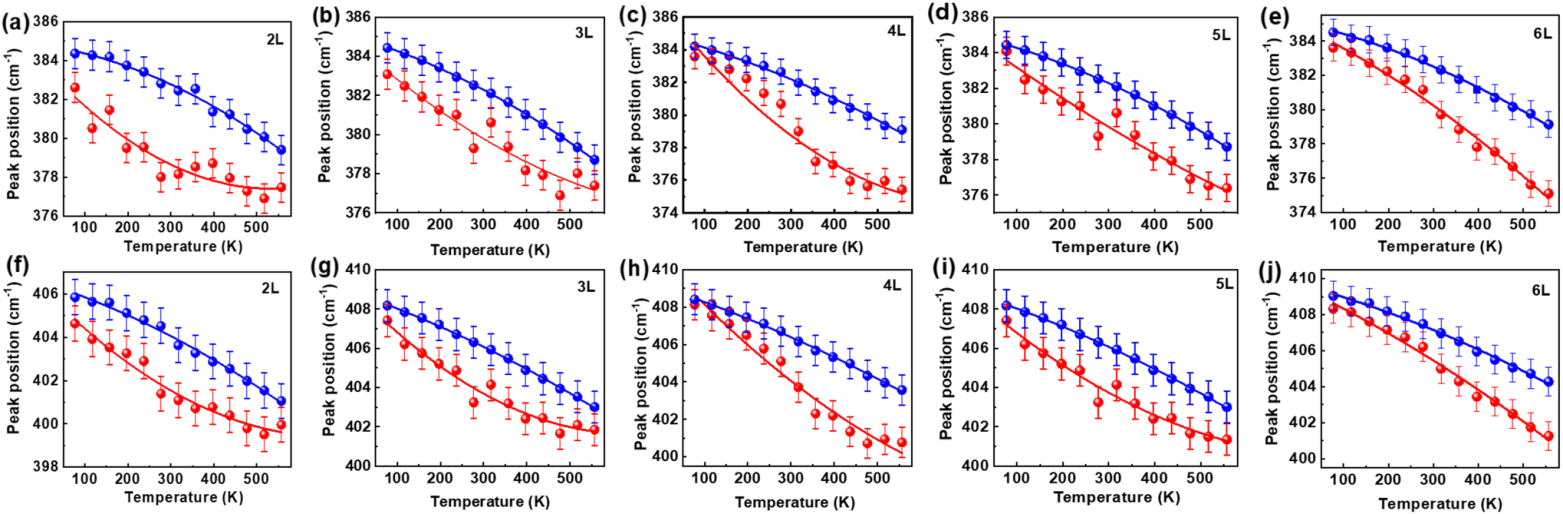
Temperature dependence of peak positions of the (**a**–**e**) E_2g_ and (**f**–**j**) A_1g_ modes for the suspended and supported MoS_2_ with different numbers of layers. The blue spheres and red spheres represent the experimental results of supported and suspended MoS_2_, respectively. The blue lines and red lines are the fitting results obtained using a second-order polynomial function of temperature.

To obtain deeper insight into the difference between suspended and supported MoS_2_, the Raman spectra were deconvoluted using a Lorentz/Gaussian mixed function. The peak positions of the E_2g_ and A_1g_ modes are plotted as a function of temperature in one figure for comparison. Figure 4 exhibits the fitting results for supported and suspended MoS_2_, in which several remarkable phenomena should be addressed, as discussed below.

First, the temperature-dependent evolutions of the supported MoS_2_ samples are similar, varying approximately linearly with increasing temperature at first sight. This suggests that the substrate effect is exerted on the MoS_2_ flakes as a whole, although the substrate is only in direct contact with the bottom layer of a MoS_2_ flake. The mechanically exfoliated MoS_2_ layer is transferred and pinned on the substrate by the van der Waals force. As the temperature changes, the biaxial tensile or compressive stress induced by the TEC mismatch increases and becomes a prominent factor that modulates the frequency shift of Raman peaks. In addition to TEC mismatch, charge transfer between the film and the substrate or through interfacial states can impact the temperature evolution of Raman peak. As Su et al. discussed that accelerated redshift of A_1g_ mode with increasing temperature is associated with the enhanced charge injection from the substrate into the film and decomposition of adsorbed contaminants^30^.

Second, the temperature-dependent evolutions of suspended MoS_2_ are very different from those of supported MoS_2_, exhibiting nonlinear behavior with increasing temperature. Moreover, the temperature dependence trends for the different suspended MoS_2_ samples differ. As discussed previously, the TEC mismatch gives rise to a Raman shift with increasing temperature. However, suspended MoS_2_, at least the part on the hole, is free of the substrate effect, suggesting that its Raman shift only originates from lattice expansion. Compared with the temperature dependence of supported MoS_2_, suspended few-layer MoS_2_ exhibits the intrinsic thermal properties of MoS_2_ as expected.

Third, the peak positions of supported MoS_2_ are higher than those of suspended MoS_2_ at each temperature, suggesting that the TEC mismatch induced compression of the crystalline lattice in supported MoS_2_. That the larger attached area of supported MoS_2_ compared with suspended MoS_2_ would introduce more strain into the MoS_2_ layer is easy to explicate. As demonstrated previously, the strain in the MoS_2_ layer is due to the TEC mismatch between the SiO_2_ substrate and MoS_2_.

The results shown in Fig. 4 demonstrate that the TEC of MoS_2_ is strongly correlated with the number of layers, which can only be obviously exhibited after isolating it the from the substrate effect.

Then, the peak positions of MoS_2_ were fitted as a function of temperature to obtain the regularities of the temperature dependence of the peak positions. First, a linear function was employed to fit temperature evolution Raman peaks for the supported and suspended MoS_2_ samples (see Fig. S2). The temperature evolution of Raman peaks for the supported MoS_2_ shows a nearly linear behavior as a function of temperature. But there is a small deviation between the experimental results and fitting curve, as exhibited in Fig. S2. On the other hand, the temperature evolution for the suspended MoS_2_ cannot be well fitted using a linear function, in which large discrepancies between the linearly fitted curves and experimental results are observed. According to previous literatures, the anharmonic effect caused by the phonon–phonon coupling leads to the nonlinear temperature-dependent behavior of the Raman peaks^28^.

Therefore, the polynomial function was adopted to fit the experimental results for the supported and suspended MoS_2_ samples instead of the linear function. All the temperature dependence trends of the *E*_*2g*_ and *A*_*1g*_ modes were fitted using a second-order polynomial function of temperature *T*,

1 $$\upomega \left(T\right)={\omega }_{0}+{\chi }_{1}T+{\chi }_{2}{T}^{2}$$

where ${\omega }_{0}$ is the frequency at 0 K and ${\chi }_{1}$ and ${\chi }_{2}$ are the first- and second-order temperature coefficients, respectively. The fitting results for supported and suspended MoS_2_ with the same thickness are plotted in Fig. 4 for comparison, and the fitting parameters are listed in Table 1. As shown in Fig. 4, the polynomial curves better fit the experimental results for the supported and suspended MoS_2_, compared with the linear curves. Remarkably, for the supported MoS_2_, the residual sum of square (RSS) for the polynomial fitting is much smaller than that for the linear fitting (see Table S1 in the Supplementary Information). This implies that the polynomial function is a better and more reasonable choice for fitting the temperature-evolution of the Raman shifts for the supported MoS_2_, although it exhibits an approximate linear trace.

**Table 1:** Temperature coefficients of the suspended and supported few-layer MoS_2_ samples with polynomial fitting to the second order.

		E_2g_ mode			A_1g_ mode		
		ω_0_	χ_1_	χ_2_	ω_0_	χ_1_	χ_2_
2L	Sus	383.835	− 0.024	2.249 × 10^–5^	406.271	− 0.020	1.490 × 10^−5^
	Sup	384.812	− 0.003	− 1.153 × 10^–5^	406.550	− 0.006	− 6.557 × 10^–6^
3L	Sus	385.948	− 0.029	2.622 × 10^–5^	409.181	− 0.026	2.334 × 10^–5^
	Sup	384.995	− 0.006	− 8.833 × 10^–6^	408.843	− 0.008	− 5.139 × 10^–6^
4L	Sus	386.710	− 0.033	2280 × 10^–5^	410.519	− 0.025	1.168 × 10^−5^
	Sup	384.884	− 0.007	− 7.127 × 10^–6^	409.096	− 0.008	− 4.157 × 10^–6^
5L	Sus	384.990	− 0.019	5.616 × 10^–6^	408.667	− 0.020	1.236 × 10^–5^
	Sup	384.214	− 0.008	− 1384 × 10^–5^	409.105	− 0.011	− 5.266 × 10^–6^
6L	Sus	384.935	− 0.013	− 9.845 × 10^–6^	409.585	− 0.012	− 6.340 × 10^–6^
	Sup	385.099	− 0.006	− 8.738 × 10^–6^	409.719	− 0.007	− 5.627 × 10^–6^

As exhibited in Table 1, the fitting parameters for suspended MoS_2_ are very different from those for supported MoS_2_. The fitting parameter *χ*_1_ for suspended MoS_2_ is one order of magnitude larger than that for supported MoS_2_. Moreover, the fitting parameters exhibit a layer number dependence. As exhibited in Table 1, for the 2L–5L MoS_2_ samples, the *χ*_2_ of suspended MoS_2_ is positive, whereas the *χ*_2_ of supported MoS_2_ is negative. Interestingly, *χ*_2_ is negative for both suspended and supported 6L MoS_2_. This occurs because of the different temperature evolutions of 6L MoS_2_ and thinner MoS_2_. One can see in Fig. 4e and j that the peak positions for the 6L suspended MoS_2_ linearly shift to low frequency, similar as the peak evolution for the supported MoS_2_. Therefore, the fitting parameters for the curves are all negative. In contrast, the shift rates of the peak positions for the 2L–5L suspended MoS_2_ vary in different temperature ranges. As exhibited in Fig. 4a–d, the peak positions shift faster in the low temperature range (< 350 K) than in the high temperature range (> 350 K). Therefore, the parameter χ_2_ is positive to better fit the experimental results. The thermal stability of MoS_2_ strongly depends on the competition between the energy barriers introduced by the MoS_2_–substrate interface and by the MoS_2_–MoS_2_ interlayer interface^44^. The few-layer MoS_2_ flake as a whole changes with increasing temperature, the influence of the intrinsic thermal expansion of MoS_2_ on the frequency shift increases as the number of layers increases. As a result, the temperature evolutions of the Raman peaks of the suspended and supported 6L MoS_2_ samples become similar. These results suggest that the thermal behavior of few-layer MoS_2_ become similar as that of bulk MoS_2_ with increasing thickness.

**Figure 5:**
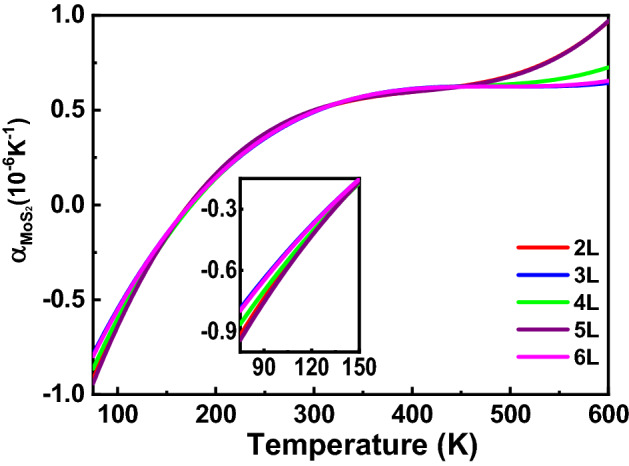
Calculated TECs of MoS_2_ with different numbers of layers. The inset figure shows a magnified view of the TECs in the temperature range of 75–150 K.

The results in Table 1 indicate that the discrepancy in the frequency shifts between the suspended and supported MoS_2_ originates from the TEC mismatch between the substrate and MoS_2_. Taking advantage of the results shown in Figs. 3, 4 and 5, the TEC of few-layer MoS_2_ can be obtained, and the details for the calculation of the TEC of few-layer MoS_2_ will be discussed in the following section.

As has been reported, the temperature-dependent Raman frequency shift ($\Delta {\omega }_{MoS2}(T)$) of freestanding MoS_2_ can be commonly attributed to the thermal expansion of the lattice ($\Delta {\omega }^{E}(T)$) and the anharmonic effect ($\Delta {\omega }^{A}(T)$), which changes the phonon self-energy^45^. $\Delta {\omega }_{MoS2}(T)$ can be expressed as

2 $$\Delta {\omega }_{MoS2}(T)=\Delta {\omega }^{E}\left(T\right)+\Delta {\omega }^{A}\left(T\right)$$

$\Delta {\omega }_{MoS2}(T)$ can be obtained using the peak position at *T* $({\omega }_{MoS2}\left(T\right))$ subtracted by the peak position at *T*_0_ = 300 K $\left({\omega }_{MoS2}\left({T}_{0}\right)\right)$.

For the thermal behavior of the supported MoS_2_, both common thermal effects and strains induced by the TEC mismatch between the substrate and MoS_2_ must be considered. As a result, the frequency shifts of supported MoS_2_ can be written as

3 $$\Delta {\omega }_{MoS2}^{S}\left(T\right)=\Delta {\omega }^{E}\left(T\right)+\Delta {\omega }^{A}\left(T\right)+\Delta {\omega }^{S}\left(T\right)$$

The E_2g_ mode arises from the in-plane relative vibration between the Mo and S atoms, which is more sensitive to the temperature-induced lattice expansion/shrinkage of 2D MoS_2_. On the other hand, the frequency shift of A_1g_ mode not only closely depends on lattice variations, but also is related with the charge transfer from the substrate to MoS_2_^46^. The electron doping effect can induce the frequency shifts of A_1g_ mode due to the strong electron–phonon interaction^47^. Consult to previous literature, the E_2g_ mode is not sensitive to the electron doping effect^47,48^. So it is assumed that the electron doping effect induced Raman shift of E_2g_ mode did not change with temperature, or the changes can be neglected. For simplicity, the doping effect induced Raman shift of E_2g_ mode is defined as a constant that is independent of temperature in this work. Thus, in the calculation of the Raman frequency differences ($\Delta {\omega }_{MoS2}(T)$) between the given temperatures and *T* = 300 K, the doping effect induced Raman shift is subtracted as a constant. So that there are still three terms in Eq. (3) when the doping effect induced Raman shift is not taken into consideration. Therefore, the $\Delta {\omega }_{MoS2}(T)$ of E_2g_ mode was calculated and employed in the following equations.

The TEC mismatch-induced frequency shift can be obtained by subtracting the intrinsic frequency shift from the frequency shift of supported MoS_2_,

4 $$\Delta {\omega }^{S}\left(T\right)=\Delta {\omega }_{MoS2}^{S}\left(T\right)-\Delta {\omega }_{MoS2}(T)$$

To apply Eq. (4), the frequency shift of freestanding MoS_2_ should be provided. However, real freestanding MoS_2_ does not exist. Therefore, Raman frequency shifts from theoretical calculations or suspended TMDs have normally been employed as those of freestanding samples^31,35^. In this work, we assume that the strain induced by the substrate effect can be neglected in the center of the suspended MoS_2_ layers, as the laser spot (1 μm) in the measurement is much smaller than the size of the hole (5 μm) below the suspended MoS_2_. Therefore, the Raman shifts of suspended MoS_2_ is adapt as the intrinsic frequency of freestanding MoS_2_ in this work.

Based on above discussion, the TEC mismatch-induced frequency shift $\Delta {\omega }^{S}\left(T\right)$ for the supported MoS_2_ can be obtained by

5 $$\Delta {\omega }^{S}\left(T\right)=\Delta {\omega }_{sup}\left(T\right)-\Delta {\omega }_{sus}\left(T\right)$$

where $\Delta {\omega }_{sup}\left(T\right)$ and $\Delta {\omega }_{sus}\left(T\right)$ are the Raman shifts of supported and suspended MoS_2_, respectively.

In addition, the contribution to the Raman frequency shift from the substrate-induced strain ($\Delta {\omega }^{S}\left(T\right)$) can be expressed as

6 $$\Delta {\omega }^{S}\left(T\right)=\beta {\int }_{{T}_{0}}^{T}[{\alpha }_{Si{O}_{2}}\left(T\right)-{\alpha }_{Mo{S}_{2}}\left(T\right)]$$

where *β *is the biaxial strain coefficient of the Raman mode and ${\alpha }_{Si{O}_{2}}\left(T\right)$ and ${\alpha }_{Mo{S}_{2}}\left(T\right)$ are the temperature-dependent TECs of SiO_2_ and MoS_2_, respectively. As has been reported, *β* depends on the number of MoS_2_ layers^49^.

As the values of ${\alpha }_{Si{O}_{2}}$ and *β*are already known from previous literatures, the temperature dependence of ${\alpha }_{Mo{S}_{2}}$ can be derived from Eqs. (5) and (6). The calculated ${\alpha }_{Mo{S}_{2}}\left(T\right)$ can be expressed using a quadratic function, and then, the ${\alpha }_{Mo{S}_{2}}\left(T\right)$ values for MoS_2_ with different numbers of layers are plotted in Fig. 5.

As presented in Fig. 5, the curves of the calculated TECs of MoS_2_ with different numbers of layers follow similar trends. Notably, the order of magnitude of the TECs is at the same level as those in previous reports^22,28^, implying the validity of the calculation methods employed in this work. For example, the TEC at room temperature observed in this work is approximately 0.5 × 10^–6^ K^−1^. Su et al. claimed that the in-plane TEC of MoS_2_ is 2.48 × 10^–6^ K^−1^ at room temperature^28^, whereas Late et al. reported a TEC of 8.2 × 10^–6^ K^−1^^22^. The discrepancy in the TECs between our results and previous publications can be attributed to the diversity in *β* employed in the calculation and whether the substrate effect is considered. Remarkably, the TECs of few-layer MoS_2_ are very close in the temperature range of 150–450 K. These results clearly suggest the feasibility of using Raman spectroscopy in the investigation of the TEC of MoS_2_, at least in the temperature range of 150–450 K.

In addition, the diversity in the TECs between the MoS_2_ with different numbers of layers is also obvious. As presented in the inset figure of Fig. 5, the TECs of few-layer MoS_2_ exhibit remarkable differences in the temperature ranges of 0–150 K and 450–600 K. Strikingly, the TEC becomes negative below 175 K. This is different from most previous reports^25,28,50,51^, in which the TEC is positive in the entire temperature range. In 2015, Wang et al. obtained a negative TEC below 31 K for monolayer MoS_2_ using first-principles calculation by taking the stiffness and charge transfer effect into consideration^52^. The ZA bending vibrations (acoustic modes) may cause negative thermal expansion in few-layer MoS_2_^52,53^. The negative value of the Grüneisen parameter for the transverse acoustic mode responds for the negative TEC^54^. The larger the absolute value of the negative Grüneisen parameter is, the larger the negative TEC. The negative TEC below 175 K observed in our work suggests a larger negative Grüneisen parameter.

Moreover, the TEC of few-layer MoS_2_ increases gently in the temperature range over 450 K, as shown in Fig. 5. This evolution of the TEC observed in our work is similar to that in previous studies^50–52^. However, the high-temperature TECs for 4L and 5L MoS_2_ exhibit a slight difference compared with the other thicknesses. Wang et al. reported that the threshold temperature for etching monolayer MoS_2_ is lower than 513 K, which is closely related with defects^44^. The abnormal behavior of the TECs for 4L and 5L MoS_2_ in the high temperature range can be attributed to the lower thermal stability due to the defects initially existed in these MoS_2_ samples. Identification of the TEC of few-layer MoS_2_ requires further experimental and theoretical studies. In the future, the temperature-dependent Raman study carried with controllable electronic doping concentration is called to deeply investigate the doping and dielectric environment effects on the frequency shifts of Raman modes, especially the A_1g_ mode.

## Conclusion

In this work, a comprehensive Raman study was carried out on supported and suspended MoS_2_ with different numbers of layers in the temperature range from 77 to 557 K. Strikingly, the temperature behaviors of the Raman frequency shift for suspended MoS_2_ are significantly different from those for supported MoS_2_. The intrinsic TECs of 2–6-layer MoS_2_ were calculated after eliminating the substrate effect. Strikingly, the TEC becomes negative below 175 K, which can be associated with the bending vibration in the MoS_2_ layer as the temperature decreases. The TEC curves of MoS_2_ with different numbers of layers follow similar evolution trends in the temperature range of 150–450 K. Compared with previous reports, the validity of the TEC obtained in this work suggests that Raman spectroscopy is a feasible tool for investigating the TEC of MoS_2_. Our results provide useful information for understanding the thermal properties of MoS_2_ and its further application in devices.

## Methods

To fabricate suspended MoS_2_ samples, a periodic hole array was first fabricated on a SiO_2_ (300 nm)/Si substrate by UV lithography and reactive ion etching technology, in which the holes were 5 μm in diameter and 2 μm in depth. MoS_2_ flakes with different thicknesses were prepared from a natural MoS_2_ single crystal using a modified mechanical exfoliation method onto the prepatterned SiO_2_/Si substrate previously cleaned by oxygen plasma.

The optical image of the suspended few-layer MoS_2_ was obtained using an Olympus BX41 microscope equipped on the micro-Raman spectrometer, Horiba Evolution HR. In the Raman spectroscopy measurements, a solid-state laser with a 532 nm wavelength was used as the excitation source. The laser beam was focused using a 100 × long-working distance objective with numeric aperture NA = 0.8, and the spot size was approximately 1 μm. To avoid significant frequency shifts induced by the local heating effect and ensure a sufficient SNR, the laser power was set at ~ 0.9 mW on the surface of the heating stage. The numbers of layers of MoS_2_ were identified using ULF Raman spectroscopy. The sample was placed inside a cryostat cell (Linkam, THMS 600), and the Raman spectra were measured in the temperature range from 77 to 557 K at an interval of 20 K.

## Supplementary Information

Supplementary Information.
