# Supplementary material for: Thermal expansion coefficient of few-layer MoS2 studied by temperature-dependent Raman spectroscopy
Source: Sci Rep. 2021 Mar 29;11:7037. doi: 10.1038/s41598-021-86479-6 (PMC8007611; doi:10.1038/s41598-021-86479-6)
Supplement: Supplementary file 1 — Supplementary Information. [file 41598_2021_86479_MOESM1_ESM.pdf]

Thermal expansion coefficient of few-layer MoS_2 studied by temperature-dependent Raman spectroscopy

Zhongtao Lin [1,2,#], Wuguo Liu [1,#], Shibing Tian[1], Ke Zhu[1], Yuan Huang[1,*], Yang Yang[1,*]

1 Beijing National Laboratory for Condensed Matter Physics, Institute of Physics, Chinese Academy of Sciences, P O Box 603, Beijing, 100190, People’s Republic of China

2 Faculty of Materials and Manufacturing, Beijing University of Technology, Beijing, 100124, People's Republic of China

* Correspondence author.

E-mail addresses: yhuang01@iphy.ac.cn (Y. Huang), yang.yang@iphy.ac.cn (Y. Yang)

Supplementary Materials

Figure S1 provides the fitting results of the room temperature spectra for the selected supported and suspended samples (2L, 4L and 6L). As the supported 2L MoS_2 exhibited asymmetric line shape, its spectrum was fitted using single peak and double peaks, and plotted in Figs. S1(a) and S1(b), respectively.

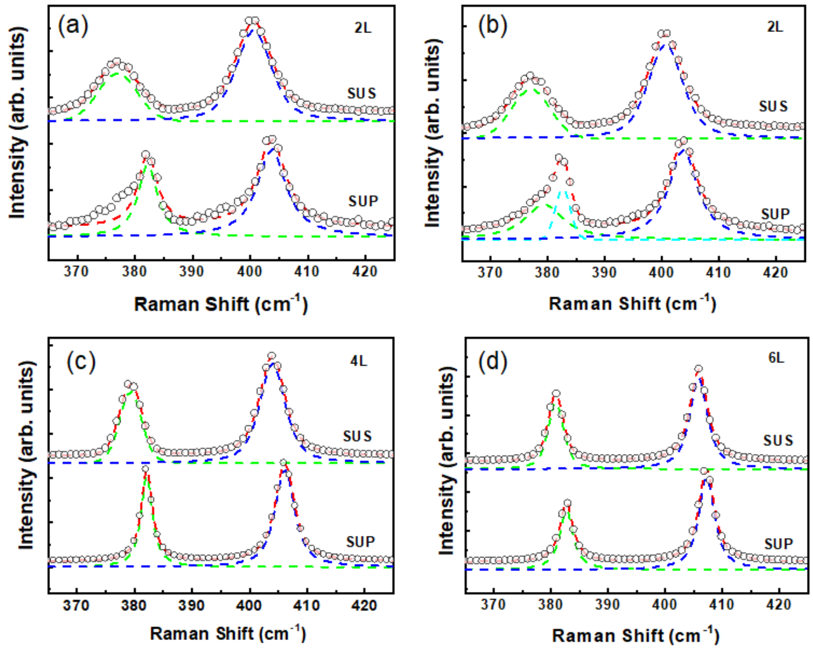

Figure S2 presents the temperature dependence of the peak positions of the E_{2g} and A_{1g} modes fitted using a linear function.

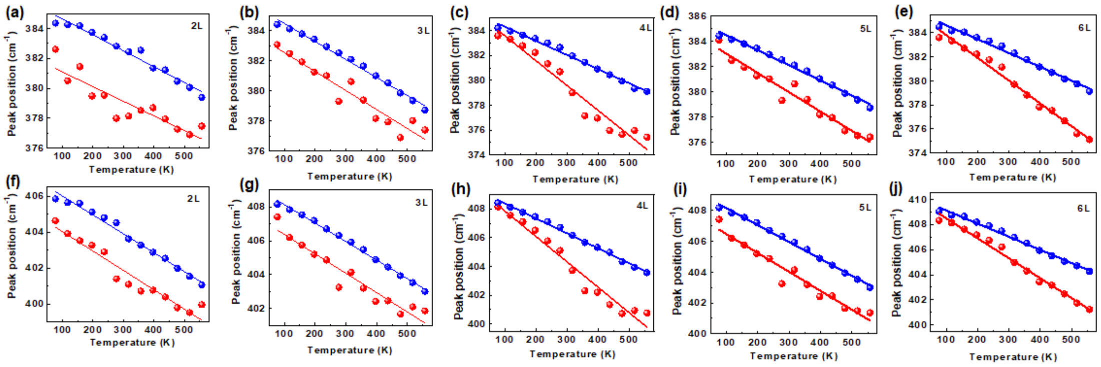

Table S1 The residual sum of square for the polynomial fitting and the linear fitting curves for the supported MoS_2 with different number of layers.

	E_{2g} mode		A_{1g} mode	
	Nonlinear fitting	Linear fitting	Nonlinear fitting	Linear fitting
1L-sub	0.13251	0.3265	0.37041	0.80722
2L-sub	0.38726	1.06904	0.27078	0.49112
3L-sub	0.01801	0.41789	0.03151	0.16684
4L-sub	0.19149	0.45184	0.06828	0.15683
5L-sub	0.01801	0.41789	0.03151	0.16684
6L-sub	0.14067	0.53203	0.14295	0.30524
